# Requirement for BUB1B/BUBR1 in tumor progression of lung adenocarcinoma

**DOI:** 10.18632/genesandcancer.53

**Published:** 2015-03

**Authors:** Honglin Chen, James Lee, Noelyn M. Kljavin, Benjamin Haley, Anneleen Daemen, Leisa Johnson, Yuxin Liang

**Affiliations:** ^1^ Departments of Molecular Biology, Genentech Inc., South San Francisco, California, USA; ^2^ Discovery Oncology, Genentech Inc., South San Francisco, California, USA; ^3^ Molecular Oncology, Genentech Inc., South San Francisco, California, USA; ^4^ Bioinformatics and Computational Biology, Genentech Inc., South San Francisco, California, USA

**Keywords:** BUB1B/BUBR1, metastasis, lung adenocarcinoma, anchorage-independent growth, anoikis

## Abstract

Lung adenocarcinoma is often discovered as metastatic disease with very poor prognosis. However, much remains unknown about the mechanisms of lung adenocarcinoma tumor progression. In this study we showed that knockdown of BUB1B/BUBR1, a critical mitotic checkpoint protein, significantly inhibited anchorage-independent growth of lung adenocarcinoma cell lines. In allograft and tail vein mouse model studies, BUB1B suppression inhibited primary tumor growth and reduced metastasis to the lung and lymph nodes, resulting in prolonged survival in both tumor prevention and tumor intervention settings. Mechanistic studies revealed that BUB1B knockdown sensitized cells to anoikis. The N-terminal region and GLEBS domain of BUB1B were required for its functions in both anchorage-independent growth and anoikis resistance, whereas the kinase domain was less critical. Overexpression of BUB1B is associated with disease progression and poor survival in human lung adenocarcinoma patients. Collectively, these data reveal a novel function for BUB1B in mediating anchorage-independent survival and growth, thereby facilitating lung adenocarcinoma dissemination during metastasis. Thus, targeting BUB1B could provide potential therapeutic benefit in suppressing metastasis and prolonging survival in lung adenocarcinoma patients.

## INTRODUCTION

Lung cancer is the leading cause of cancer-related mortality worldwide, resulting in more than a million deaths per year [1]. Lung adenocarcinoma is the most common subtype, representing over 40% of diagnosed lung cancer. Identification of driver mutations that frequently occur in subsets of lung adenocarcinoma has brought significant advances in molecular diagnostics and targeted therapies for this disease. However, about half of lung adenocarcinoma harbors no known oncogenic drivers [2, 3]. Furthermore, oncogenic driver mutations like *KRAS* and loss of function mutations and deletions in tumor suppressor genes such as *TP53* are difficult to exploit therapeutically. Importantly, while lung adenocarcinoma is often discovered as locally advanced or metastatic disease with an average 5-year survival rate of ~15% [4], the mechanisms mediating tumor progression and metastasis remain largely uncharacterized. Indeed, a recent mapping of somatic alterations to cancer hallmarks illuminates many gaps in the understanding of the somatic underpinnings of lung adenocarcinoma [5]. Therefore, knowledge of additional genomic changes in lung adenocarcinoma, especially those involved in tumor metastasis, is needed to further guide diagnosis and treatment.

Activating mutations in the *KRAS* oncogene and inactivation of the *TP53* tumor suppressor gene are the most common genetic abnormalities identified in human lung adenocarcinoma, occurring in ~30% and 50% of the patients, respectively [6]. Well-established genetically engineered mouse models that utilize conditional alleles of oncogenic *Kras*, either alone or in combination with *Trp53* mutation, recapitulate the stepwise progression of lung adenocarcinoma and phenocopy human therapeutic responses to standard-of-care treatment regimens [7]. Many studies have been performed employing these mouse tumor models and cell lines derived from them to investigate the molecular mechanisms of lung adenocarcinoma initiation and progression [8, 9]. Here, using several such cell lines, we uncovered a novel role for BUB1B, a mitotic checkpoint kinase, in regulating anchorage-independent growth, anoikis and metastasis. These data in combination with clinical human patient data identify BUB1B as a new molecular target that plays a critical role in tumor progression of lung adenocarcinoma.

## RESULTS

### BUB1B is required for anchorage-independent growth of lung adenocarcinoma cell lines

**Figure 1:**
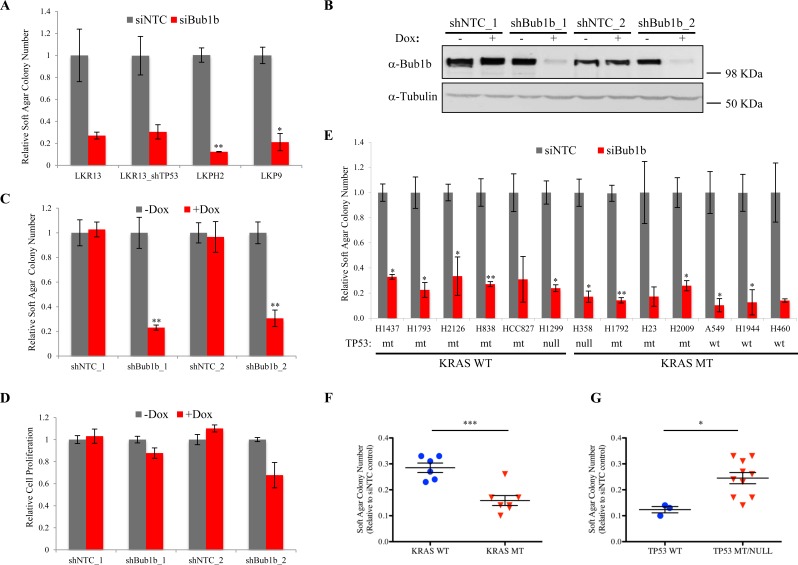
BUB1B knockdown inhibits anchorage-independent growth of lung adenocarcinoma cell lines A. Soft agar colony formation of mouse lung adenocarcinoma cell lines transfected with siNTC or mouse *Bub1b* siRNA SMARTpool. Colony numbers were shown as relative values normalized to siNTC controls. Mean±SEM was calculated from two independent experiments. The t-test p-values for siNTC vs siBub1b in LKR13 and LKR13_shTP53 cells were 0.09 and 0.07, respectively. B. BUB1B protein levels in LKPH2 cells expressing inducible shNTC or shBub1b 72 hours after induction with 100ng/ml doxycycline. C. Soft agar colony formation of LKPH2 cells from B. Colony numbers were shown as relative values normalized to no doxycycline treatment controls. Mean±SEM was calculated from three independent experiments. D. Cell proliferation of LKPH2 cells from B measured by CellTiterGlo. Values were normalized to no doxycycline treatment controls. Mean±SEM was calculated from two independent experiments. E. Soft agar colony formation of human lung adenocarcinoma cell lines transfected with siNTC or human *BUB1B* siRNA SMARTpool. Colony numbers were shown as relative values normalized to siNTC controls. Mean±SEM was calculated from two (HCC827, H23 and H460) to three independent experiments. The t-test p-values for siNTC vs siBub1b in HCC827, H23 and H460 cells were 0.1, 0.09 and 0.07, respectively. WT/wt, wild type. MT/mt, mutant. F and G. Cell lines in E were grouped based on their *KRAS* (F) or *TP53* (G) status. *** indicates p<0.001, ** p<0.01, * p<0.05.

To discover new therapeutic targets mediating progression of lung tumors driven by oncogenic *Kras* and *Trp53* deletion, we conducted an RNAi screen targeting 713 unique mouse kinases using a soft agar colony formation assay (Figure S1). The initial screen was conducted in the *Kras^LSL-G12D /+^* lung tumor derived LKR13 cell line expressing a *Trp53* targeting short hairpin shRNA (LKR13_shTP53) that reduced the *Trp53* mRNA level by ~50% (data not shown). BUB1B was identified as one of the top hits, knockdown of which dramatically reduced anchorage-independent growth (hereafter referred to as AIG) but only modestly decreased cell proliferation in two-dimensional (2D) liquid culture. We validated this observation with siRNAs in multiple mouse lung adenocarcinoma cell lines expressing oncogenic *Kras* with varied *Trp53* status (Figure 1A, Table S1). We subsequently generated inducible stable cell lines targeting *Bub1b* using doxycycline-regulated shRNAs (shBub1b_1 and shBub1b_2) in the LKPH2 cell line *Kras^LSL-G12D^* that carries a *^/+^/Trp53^Flox/−^*allele. Seventy-two hours after induction with doxycycline, BUB1B protein level in these cell lines was significantly reduced relative to the non-targeting control shRNA (shNTC_1 and shNTC_2) expressing cell lines (Figure 1B), and this correlated with significant decreases in AIG (Figure 1C and S2). Cell proliferation in 2D liquid culture was again only modestly decreased (Figure 1D). We further examined the effect of BUB1B knockdown in multiple human lung adenocarcinoma cell lines (Table S1). The endogenous BUB1B protein was knocked down >80% in all of the human cell lines tested (Figure S3). Regardless of their *KRAS* and *TP53* status, all cell lines demonstrated substantially reduced AIG (Figure 1E). Notably, cell lines harboring *KRAS* mutations were more sensitive to BUB1B knockdown than those with wild-type *KRAS* (Figure 1F). Very few human lung adenocarcinoma cell lines with wild-type *TP53* were capable of AIG, however three cell lines that were permissive were more sensitive to BUB1B knockdown compared to those harboring *TP53* alterations (Figure 1G).

### The N-terminal region and GLEBS domain of BUB1B are required for anchorage-independent growth of lung adenocarcinoma cell lines

**Figure 2:**
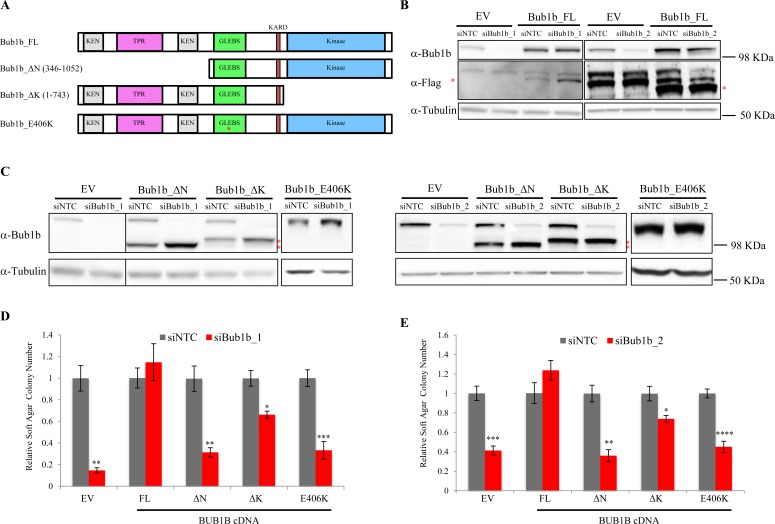
Rescue of anchorage-independent growth in LKPH2 cells with ectopic expression of RNAi-resistant mouse *Bub1b* cDNAs A. Schematic representation of the mouse *Bub1b* cDNAs used in this study. Bub1b_FL, full length *Bub1b* cDNA. Bub1b_∆N, N-terminal truncated *Bub1b* cDNA. Bub1b_∆K, kinase domain deleted *Bub1b* cDNA, Bub1b_E406K, full length *Bub1b* cDNA containing a point mutation (indicated by the red asterisk) in the GLEBS domain that interrupts BUB3 binding and kinetochore localization. The conserved functional motifs are indicated. B and C. BUB1B protein levels in LKPH2 cells stably expressing the indicated cDNAs 72 hours after transfection with siNTC or the indicated siBub1b. Red asterisks indicate the flag-tagged full length (B) or truncated (C) BUB1B. EV: empty vector. D and E. Soft agar colony formation of LKPH2 cells stably expressing the indicated cDNAs transfected with siNTC or the indicated siBub1b. Colony numbers were shown as relative values normalized to siNTC controls. Mean±SEM was calculated from three to five independent experiments. **** indicates p<0.0001, *** p<0.001, ** p<0.01, * p<0.05.

Several BUB1B functional motifs have been described to play critical roles in spindle assembly checkpoint (SAC) signaling and stable attachment of kinetochores to spindle microtubules during mitosis [10-12]. In the N-terminal region, two Lys-Glu-Asn (KEN) box motifs are required for Cdc20 binding and anaphase-promoting complex/cyclosome (APC/C) inhibition. A three tetratricopeptide repeat (TPR) within this region also contributes to Cdc20 binding as well as the recruitment of BUB1B to kinetochores. In the middle region, a BUB3-binding motif (GLEBS) is essential for BUB1B localization to kinetochores during mitosis, and the kinetochore attachment regulatory domain (KARD) promotes proper kinetochore-microtubule attachment through its interaction with PP2A-B56α. Functions of the C-terminal kinase domain in mitosis are unclear. Most studies indicate that it is dispensable for SAC signaling but may be important for correct kinetochore-microtubule attachment. In addition, the kinase domain may regulate BUB1B protein stability. To confirm that the observed effect on AIG was due to BUB1B knockdown as well as to delineate the regions of BUB1B required for AIG, we performed rescue experiments in LKPH2 cells stably expressing full-length, domain-deleted or mutated, RNAiresistant isoforms of murine *Bub1b* cDNA (Figure 2A). Seventy-two hours after transfection with a non-targeting control (siNTC) or *Bub1b* targeting (siBub1b_1 or siBub1b_2) siRNA, all forms of ectopic *Bub1b* cDNAs were stably expressed in the presence of siBub1b while the endogenous *Bub1b* was down-regulated (Figures 2B and 2C). In comparison to the empty vector control, expression of a full-length wild-type *Bub1b* cDNA (Bub1b_FL) fully reversed the reduction of AIG caused by knockdown of the endogenous *Bub1b*. In contrast, expression of an N-terminal truncated *Bub1b* cDNA (Bub1b_∆N) or a full-length *Bub1b* cDNA containing the E406K mutation in the GLEBS domain that disrupts BUB3 binding and kinetochore localization (Bub1b_Ε406Κ) failed to rescue, whereas expression of a kinase domain-deleted *Bub1b* cDNA (Bub1b_∆K) rescued ~60% of the reduction in AIG (Figures 2D-2E). These results confirm that BUB1B is required for AIG of lung adenocarcinoma cell lines and indicate that the N-terminal region and GLEBS domain are indispensable for this function, whereas the kinase domain is less essential.

### BUB1B knockdown inhibits tumor growth and metastasis, and prolongs survival

**Figure 3:**
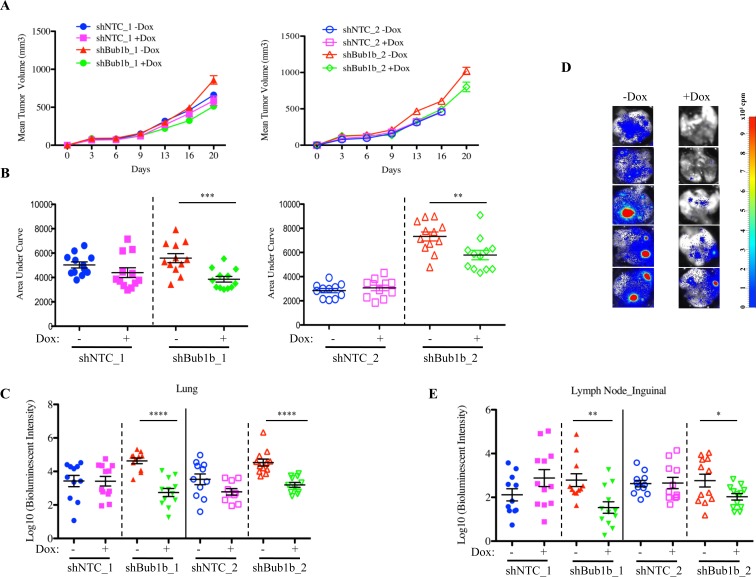
BUB1B knockdown inhibits tumor growth and metastasis in LKPH2 allografts A. Tumor volumes in mice implanted with LKPH2 cells expressing inducible shNTC or shBub1b. Mice implanted with shNTC_2 expressing LKPH2 cells were inadvertently euthanized for *ex vivo* bioluminescence imaging before tumor volume measurement on day 20. n=10-12/group. Mean±SEM is shown. B. Comparison of tumor growth using an AUC measurement between day 0 and day 20 (day 16 for shNTC_2). Smaller AUC values represent slower tumor growth. C. Quantification of lung metastases in mice from A and B measured by *ex vivo* bioluminescence imaging at the end of study. D. Representative *ex vivo* bioluminescent images of lung from mice implanted with LKPH2 cells expressing shBub1b_1 at the end of study. E. Quantification of inguinal lymph node metastases measured by *ex vivo* bioluminescence imaging at the end of study. **** indicates p<0.0001, *** p<0.001, **p<0.01, *p<0.05.

To investigate the effect of BUB1B knockdown *in vivo*, we implanted the LKPH2 inducible shRNA stable cell lines subcutaneously into nude mice and measured primary tumor volumes as well as metastases in the lung and lymph nodes. At the end of study (day 20), BUB1B levels remained substantially reduced (~60-70%, Figure S4), which correlated with a modest reduction in primary tumor growth (Figures 3A and 3B). In addition, metastases in the lung and inguinal lymph nodes, as determined by the intensity of bioluminescent signals, were decreased following knockdown of BUB1B (Figures 3C-3E).

**Figure 4:**
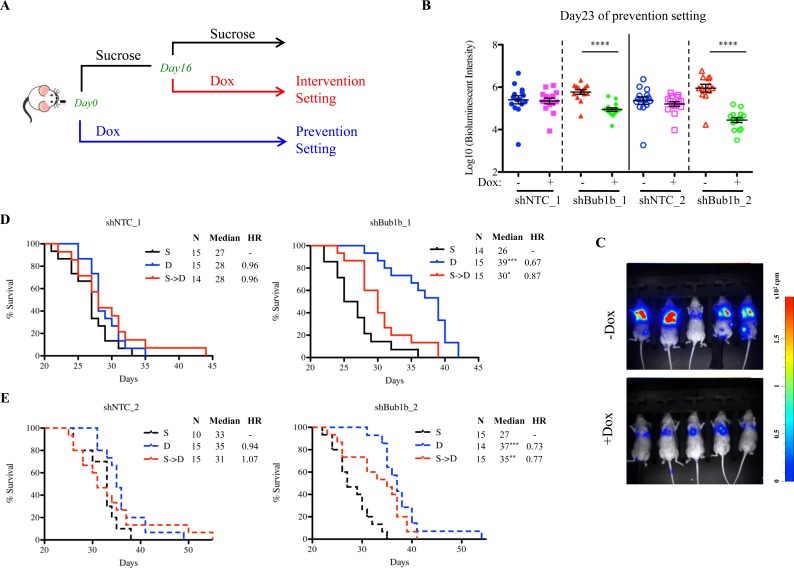
BUB1B knockdown prolongs survival in LKPH2 tail vein injection mouse model A. Schematic design of the study. B. Quantification of whole body tumor burden by *in vivo* bioluminescence imaging at day 23 post injection in the tumor prevention setting. C. Representative *in vivo* bioluminescent images of mice at day 23 post injection with LKPH2 cells expressing shBub1b_2 in the tumor prevention setting. D and E. Kaplan-Meier survival plots of mice injected with LKPH2 cells expressing inducible shNTC (left panel) or shBub1b (right panel). n=10-15/group. S: mice received 5% sucrose water throughout the study. D: mice received 5% sucrose water containing doxycycline throughout the study. S→D: mice received 5% sucrose water for the first 16 days after injection and switched to 5% sucrose water containing doxycycline for the remainder of the study. **** indicates p<0.0001, *** p<0.001, ** p<0.01, * p<0.05. HR, hazard ratio.

We further evaluated the efficacy of BUB1B knockdown on survival in two different settings using a tail vein injection model (Figure 4A). In the tumor prevention setting, BUB1B knockdown was initiated at the time of injection of LKPH2 cells. This resulted in significantly slowed systemic tumor growth as shown by bioluminescent quantification and representative images of tumor burden at day 23 (Figures 4B and 4C), which correlated with prolonged overall survival (Figures 4D and 4E). In the tumor intervention setting, BUB1B knockdown was initiated 16 days after LKPH2 cell injection, a time when systemic tumors were well established (Figure S5). Here, tumor growth was still slowed down (data not shown), and a significant overall survival benefit was similarly observed (Figures 4D and 4E). It is important to note that the impact on overall survival was likely diminished at later time points in both settings as BUB1B levels were found to only be reduced by ~15-20% at the end of these studies (data not shown).

### BUB1B knockdown sensitizes LKPH2 cells to anoikis

**Figure 5:**
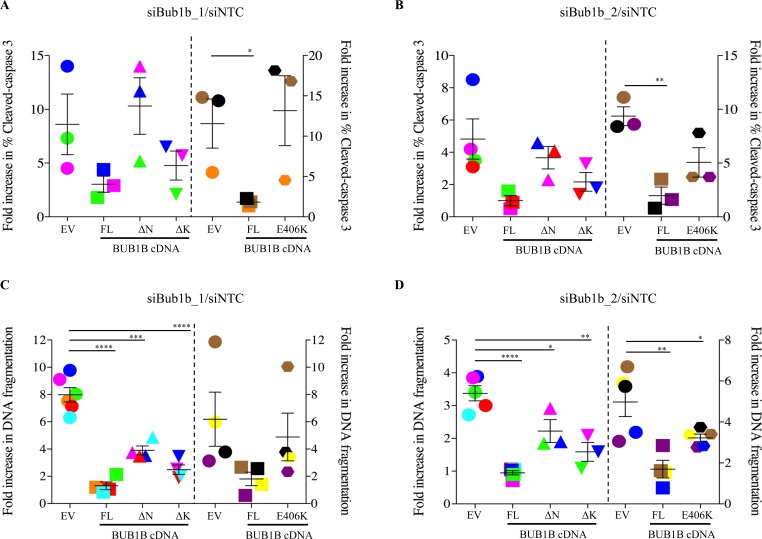
BUB1B knockdown enhances anoikis LKPH2 cells expressing the indicated *Bub1b* cDNAs were transfected with siNTC or the indicated siBub1b for 72 hours and plated onto ULA plates. Cells were collected 18 hours later for analyses of cleaved caspase-3 (A and B) and DNA fragmentation (C and D). Fold increases in the percentage of cleaved caspase-3 and DNA fragmentation were calculated as siBub1b/siNTC and (OD405-490nm) /(OD405-490nm), respectively. Mean±SEM was calculated from three to six independent siBub1b siNTC experiments. Data points with the same color were from the same independent experiment. Statistical significance was determined by t-test. **** indicates p<0.0001, *** p<0.001, ** p<0.01, * p<0.05.

Collectively, the above results suggest that BUB1B is important for anchorage-independent growth and metastasis to distant sites. To better understand the role of BUB1B in tumor metastasis, we asked which step(s) during this process was impacted by BUB1B expression. We first conducted several *in vitro* migration and invasion assays in LKPH2 cells but found that BUB1B knockdown had no effect on either process (data not shown). Anoikis is a normal cell death program initiated when cells detach from their surrounding extracellular matrix (ECM). It is known that metastatic tumor cells acquire the ability to escape from anoikis (hence named anoikis-resistant) so that they can survive in a detached state, disseminate throughout the body and invade distant organs [13]. Since reduced BUB1B expression has been associated with decreased tumor metastasis in our animal studies, we interrogated whether BUB1B knockdown would sensitize cells to anoikis, thereby explaining, at least in part, the observed inhibition of tumor metastasis. Knockdown of BUB1B in LKPH2 cells that had been grown in suspension resulted in significantly increased levels of both cleaved caspase-3 (Figures 5A and 5B), a key mediator of anoikis, and DNA fragmentation as a result of detachment-induced apoptosis (Figures 5C and 5D). These increases were almost completely reversed by overexpressing Bub1b_ FL. In contrast, overexpressing Bub1b_∆K reversed on average ~60-70% of the increases, whereas overexpressing Bub1b_∆N and Bub1b_E406K reversed on average ~ 40% and 30% of the increases, respectively (Figures 5A-5D). Similar to AIG, these results demonstrate that BUB1B expression is required for anoikis resistance, and that the N-terminal region and GLEBS domain are more critical than the kinase domain for this function.

### Overexpression of BUB1B is associated with disease progression and poor survival in human lung adenocarcinoma patients

**Figure 6:**
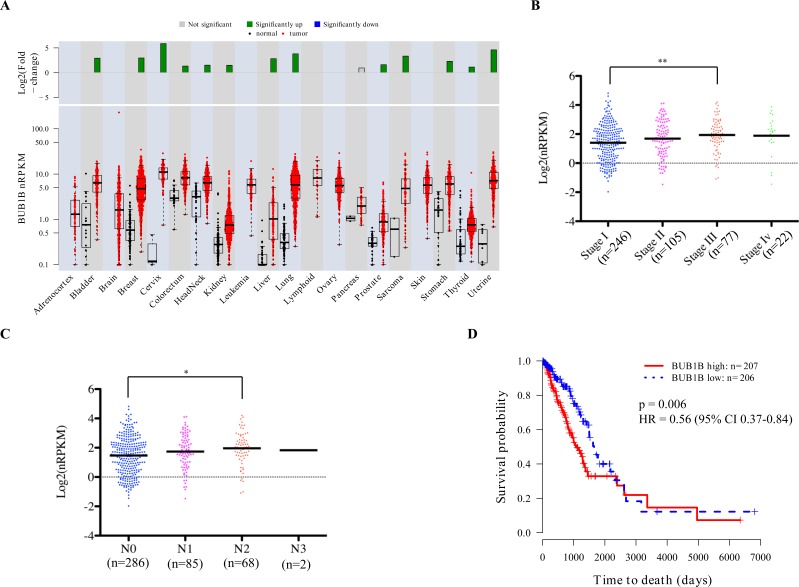
Overexpression of *BUB1B* is associated with poor overall survival in human lung adenocarcinoma patients A. *BUB1B* mRNA levels in multiple cancer types and their corresponding normal tissues from TCGA. nRPKM expression levels are shown in the boxplot on a log2-transformed scale, with display of the median expression level. The box represents 25-75% percentile. The fold-change between the mean tumor and the mean normal tissue expression is shown in the barplot on a log2-transformed scale. Significance was determined using a two-tailed t-test, with green indicative of a significant up-regulation in tumor samples (p< 0.05). B and C. *BUB1B* expression versus tumor stage (B) or lymph node metastasis (C) in TCGA lung adenocarcinoma clinical samples. One-way ANOVA test followed by Tukey's multiple comparison test was performed to determine significance. ** indicates p<0.01, * p<0.05. D. Kaplan-Meier plot for overall survival in TCGA lung adenocarcinoma clinical samples, with patients stratified into BUB1B high (*BUB1B* expression above median; red solid line) and BUB1B low (*BUB1B* expression below median; blue dotted line). CI, confidence interval.

To determine the clinical relevance of our findings, we analyzed *BUB1B* expression in human patient samples from The Cancer Genome Atlas (TCGA) project. *BUB1B* is significantly overexpressed in a majority of human cancers (Figure 6A). Correspondingly, we found that knockdown of BUB1B also significantly reduced AIG in human cell lines derived from several cancer types other than lung cancer (Figure S6). In human lung adenocarcinoma patients, higher levels of *BUB1B* expression were found in later stages of disease (Figure 6B) and lymph node metastases (Figure 6C). Higher levels of *BUB1B* expression also correlated with reduced overall survival in these patients (Figure 6D). Consistent with our data from animal model studies, these results indicate that high level expression of *BUB1B* may promote more advanced and metastatic disease.

We further examined genomic changes at the *BUB1B* locus in patient samples from the TCGA lung adenocarcinoma data set. We found that while *BUB1B* was overexpressed in 72% (351/489) of tumors compared to normal lung tissue, only 2.6% had somatic mutations and amplifications were rare, suggesting that *BUB1B* overexpression in human lung adenocarcinomas is likely to be regulated either transcriptionally or post-transcriptionally.

We also investigated the association of *BUB1B* expression with *KRAS* and *TP53* mutational status in the TCGA lung adenocarcinoma data set. We found no correlation between *BUB1B* expression levels and somatic *KRAS* mutation status (Figure S7A); however, *BUB1B* expression levels were significantly higher in lung adenocarcinomas harboring somatic *TP53* mutations (Figure S7B).

## DISCUSSION

Here we identify a novel role for BUB1B in lung adenocarcinoma progression. Specifically, BUB1B is required for anchorage-independent growth in soft agar and anoikis resistance in suspension cultures of lung adenocarcinoma cell lines. Mice implanted with cell lines in which *BUB1B* expression was knocked down by RNAi exhibited reductions in primary tumor growth and metastasis to distant sites as well as prolonged overall survival. In addition, overexpression of *BUB1B* was associated with disease progression and poor overall survival in human lung adenocarcinoma patients.

Given the critical role of BUB1B in mitotic checkpoint signaling and chromosome congression, impairment in BUB1B often results in aneuploidy and chromosomal instability, which can contribute to increased cancer incidence. Consistent with this, *Bub1b^+/−^*mice have been shown to be more cancer-prone compared to wild-type mice [14, 15]. In addition, mutations that reduce BUB1B expression in humans lead to cancer-susceptible mosaic variegated aneuploidy syndrome [16]. Interestingly, though, higher-level BUB1B expression seems to be a much more frequent event in a wide variety of human cancers and has been described as a prognostic marker for tumor recurrence and disease progression [17-27]. Mechanistically, little is known regarding how increased BUB1B expression may contribute to tumor progression. Our report is the first to identify a role for BUB1B in lung adenocarcinoma and to indicate that anchorage-independent growth and anoikis resistance may be a potential mechanism by which this occurs in lung adenocarcinoma.

BUB1B plays a central role in spindle assembly checkpoint signaling and stable attachment of kinetochores to spindle microtubules [10-12]. As such, disruption of BUB1B function often results in mitotic catastrophe. Several reports have shown that knockdown of BUB1B significantly reduced the viability of tumor cells and genetically transformed cells in 2D culture by triggering lethal chromosomal instability [17, 28]. In contrast to these findings, we found that our mutant *Kras*-driven lung adenocarcinoma cell lines were viable in 2D culture following BUB1B knockdown. Consistent with this finding, DNA content analysis revealed no substantial change in these cells' cell cycle profiles upon BUB1B knockdown (data not shown). However, BUB1B knockdown did significantly impair their survival and growth under anchorage-independent conditions. While dissecting the role of BUB1B plays in facilitating anchorage-independent survival and growth, we observed a similar pattern to that previously described for mitotic checkpoint functions -i.e., the N-terminal region and GLEBS domain are critical, whereas the kinase domain is less important [12, 17]. Therefore, one possible explanation is that while BUB1B function can be dispensable for mitotic progression of lung adenocarcinoma cells in 2D culture, loss of proper attachment from the ECM under anchorage-independent conditions *in vitro* and during tumor metastasis *in vivo* triggers a mitotic stress that renders cells more dependent on BUB1B activity. It has been reported that spindle orientation in cultured mammalian cells is influenced by the ECM [29], and that BUB1B overexpression is significantly correlated with a higher rate of cell proliferation in some human cancers [18, 23, 27]. However, the mechanisms by which increased BUB1B expression mediates tumor progression are likely to be complex as BUB1B overexpression is not always associated with increased cell proliferation in human cancers and has been found in nondividing cells as well [20, 25]. BUB1B protein is rarely detected in normal human tissues except a few that contain a substantial population of mitotic cells. In these tissues, BUB1B protein is seen with an expected nuclear localization in mitotic cells [20, 23, 30]. In contrast, in human cancer tissues, overexpressed BUB1B protein is predominantly localized in the cytoplasm [18-20, 22-25, 27]. The dissociation between BUB1B overexpression and cell proliferation in some human cancers, and the change of BUB1B protein localization during malignancy could imply that an alternative pathway other than, or in addition to, mitotic checkpoint signaling is involved in tumor progression. Cell cycle profiles and more detailed analyses to pinpoint the exact sequences and associated signaling pathways for cells grown under anchorage-independent conditions are needed to delineate the mechanisms by which BUB1B functions under these conditions and during tumor metastasis. Understanding these mechanisms will advance our knowledge of the diverse biological pathways regulated by BUB1B as well as new therapeutic opportunities that targeting BUB1B may bring.

We observed that while BUB1B knockdown reduced anchorage-independent growth of all human lung adenocarcinoma and colon cancer cell lines that we tested, those harboring *KRAS* mutations were slightly more sensitive. Oncogenic *KRAS* does not seem to regulate BUB1B expression as there was no difference in BUB1B protein levels between wild-type and mutant *KRAS* lung adenocarcinoma cell lines (data not shown) or *BUB1B* mRNA levels in lung adenocarcinoma patient samples. Rather, oncogenic *KRAS* may trigger certain conditions, such as previously described “mitotic-stress” [17, 31], which renders these cells more dependent upon BUB1B activity. In contrast, *TP53* status did not have a consistent effect on AIG reduction upon BUB1B knockdown in human and mouse lung adenocarcinoma cell lines. The relationship between *BUB1B* expression and *TP53* mutation is also unclear. While lung adenocarcinoma patients harboring a somatic *TP53* mutation or null allele express higher levels of *BUB1B* mRNA, there is no clear difference in BUB1B protein levels between wild-type and mutant/null *TP53* human and mouse lung adenocarcinoma cell lines (data not shown). Nevertheless, these data indicate that BUB1B expression promotes tumor progression irrespective of *KRAS* and *TP53* status.

In summary, we have elucidated a previously unknown function of BUB1B in progression of lung adenocarcinoma through mediating anchorage-independent survival and growth. This finding suggests that overexpression of BUB1B may serve as a predictive marker for lung adenocarcinoma disease progression and provide a new potential targeting area for inhibiting metastasis and prolonging survival in lung adenocarcinoma patients. Moreover, our finding that BUB1B knockdown inhibited metastasis and prolonged overall survival, even in settings with established tumors, suggests that therapeutic strategies targeting BUB1B may prove efficacious as single agents. This efficacy may be further enhanced in combination with therapeutic agents that significantly shrink primary tumors. Finally, *BUB1B* is overexpressed in the vast majority of human cancers and knockdown of BUB1B also significantly reduced AIG in cell lines derived from several other cancer types in addition to lung adenocarcinoma. These data suggest that BUB1B may play a universal role in the progression of multiple cancer types and thus targeting BUB1B could potentially have a broad application. However, it remains to be determined whether these cancers share a similar mechanism of action from *BUB1B* overexpression as lung adenocarcinoma.

## MATERIALS AND METHODS

### Cell lines, RNAi and antibodies

See Supplemental Table S1-S3 for lists of these reagents. Mouse lung adenocarcinoma cell lines were established from lung tumors from genetically engineered mouse models [8, 9, Genentech unpublished data]. Human lung adenocarcinoma cell lines were obtained from the Genentech Cell Line Repository. All cell lines were tested for mycoplasma and authenticated by DNA sequencing and/or PCR analysis upon receipt, cryopreserved and passaged for no more than 2 months upon resuscitation. Mouse and human cell lines were reverse transfected using Dharmafect 4 (Dharmacon) and Lipofectamine RNAimax (Life Technologies), respectively, with 100nM siRNA for 72 hours before assays were performed. Complementary double-stranded shRNA oligonucleotides were inserted into a tetracycline-inducible lentiviral vector as previously described [9].

### cDNA constructs

A lentiviral vector containing a luciferase-internal ribosomal entry site – DsRed expression cassette was used to generate stable LKPH2 cell lines for *in vivo* bioluminescence imaging. RNAi-resistant mouse wild-type and mutant *Bub1b* cDNAs were chemically synthesized (Life Technologies) and cloned into the pReceiver-Lv141 lentiviral vector (GeneCopoeia).

### Generation of stable cell lines

shRNA and cDNA lentiviral expression vectors were cotransfected with pCMV-VSVG and pCMV-dR8.9 plasmids into 293T cells using the Lipofectamine 2000 reagent (Life Technologies). Forty-eight hours after transfection viral supernatants were collected and used to infect LKPH2 cells in the presence of 8μg/mL polybrene (EMD Millipore Corp). Cells with stable integrants were selected either with 14μg/mL puromycin for 10 days or by collecting fluorescent cells using FACS. Stable cell lines generated by puromycin selection were maintained in medium containing 10μg/ml puromycin.

### Immunoblotting

Protein was extracted in lysis buffer (50mM HEPES PH7.4, 100mM NaCl, 50mM NaF, 5mM β-glycerophosphate, 2mM EGTA, 1mM Na _3_ VO _4_, 1% Triton-X 100) containing complete protease inhibitors (Roche) and quantified using BCA protein assay (Pierce). Protein was separated on 4-12% Tris/glycine gels (Invitrogen) and transferred to nitrocellulose membranes (Invitrogen), blocked with 3% milk in TBST for 30 minutes, then incubated with primary antibody overnight at 4^0^C. Membranes were then washed and incubated with appropriate HRP conjugated secondary antibodies for 1 hour, washed and detected with ChemiGlow West Chemiluminescent Substrate (Pierce). Luminescent signal was acquired using an Alpha-Innotech chemiluminescent imager (ProteinSimple).

### Mouse kinome siRNA screen and hit prioritization

LKR13_shTP53 cells were transfected in quintuplicate in 96-well plates with the Dharmacon siGENOME SMARTpool siRNA library targeting 713 mouse kinases. A non-targeting siGENOME siRNA SMARTpool (siNTC) was used as negative control. An ON-TARGETplus (OTP) siRNA SMARTpool targeting mouse *Kras* (catalog# L-043846-01) and a mouse *miR34b* microRNA mimic (catalog# C-310485-07) were used as positive controls. Seventy-two hours later, cell number in each well was quantified by the CellTiter-Glo luminescent cell viability assay (Promega). Counts from duplicate wells were used to determine cell proliferation. Cells from the remaining triplicate wells were plated into 96-well plates for soft agar colony formation. Colony numbers were scored 7 days later and normalized by the cell numbers plated. To identify targets that were most relevant to cellular transformation but not cytotoxic (and thus would likely be missed by a cell proliferation or viability based screening method), we prioritized hits whose knockdown caused a modest reduction in cell proliferation (defined as 30% or less compared to siNTC) and a significant reduction in soft agar colony formation (defined as 55% or higher compared to siNTC). These hits underwent further validation using Dharmacon's OTP siRNA SMARTpools followed by siRNA pool deconvolution in LKR13_shTP53 and LKPH2 cells. This process generated 5 top hits for further investigation.

### Cell proliferation assay

Cell proliferation was measured by the CellTiter-Glo luminescent cell viability assay in triplicates. Briefly, 100μl of CellTiter Glo reagent was added to each well of cells cultured in 100μl media in a 96-well plate. Plates were shaken gently in room temperature for 10 minutes and luminescent signals were quantified using a Victor3 luminometer (PerkinElmer) with an integration time of 0.1 second.

### Soft agar colony formation assay

Cells were cultured in an assay layer of 0.4% noble agar (BD) on top of a base layer of 0.6% noble agar. 10004000 cells were seeded per well in 6-8 repeats in a 96-well plate. Plates were scanned and analyzed after 7-21 days using the GelCount^TM^ imaging system (Oxford Optronix).

### Anoikis analysis

Anoikis was assayed by measuring levels of cleaved caspase-3 and DNA fragmentation in cells that had been grown in ultra-low attachment (ULA) plates (Corning Incorporated) for 18 hours. To measure levels of cleaved caspase-3, cells were fixed in 4% paraformaldehyde at 37°C for 10 minutes, then permeabilized in 90% methanol on ice for 30 minutes, rinsed and blocked with incubation buffer (0.5% BSA in PBS) at room temperature for 10 minutes. Cells were then incubated with anti-cleaved caspase-3 (Asp175) Pacific Blue conjugated antibody for 3 hours at room temperature, washed with incubation buffer followed by PBS, resuspended in PBS and analyzed with a BD LSR-II flow cytometer (BD Biosciences). DNA fragmentation was quantified using the Cell Death Detection ELISA^PLUS^ kit (Roche Diagnostics) according to manufacturer's instruction.

### Mouse models

For allograft tumor model, 5×10^6^ cells were implanted subcutaneously in the right flank of female athymic *nu/nu* mice of 6-8 weeks old (Harlan Laboratories). Mice received 5% sucrose water (−Dox) or 5% sucrose water containing 0.15mg/ml doxycycline (+Dox) throughout the study. Tumor volumes were measured twice a week using digital calipers. At the end of study, mice were subjected to *ex vivo* bioluminescence imaging and tumors were harvested for Western blot. Area under curve (AUC) measurement between day 0 and day 20 (day 16 for shNTC_2) was calculated for each animal using the Prism software. T-test was performed to determine statistical significance between groups treated with sucrose versus doxycycline for each cell line.

For tail vein injection tumor model, mice were injected intravenously with 3×10^4^ cells. Tumor progression was monitored weekly by *in vivo* bioluminescence imaging. Mice were weighted twice a week and monitored regularly for survival. In the tumor prevention setting, 0.03-0.05mg/ml doxycycline treatment was started at the time of injection. In the tumor intervention setting, tumors were allowed to establish for 16 days after cell injection. Mice with similar tumor burden (as determined by bioluminescent intensity) were then equally distributed into sucrose versus doxycycline cohorts, and 0.1-0.15mg/ml doxycycline treatment was initiated. Tumors were collected at the end of study for Western blot. Survival curves were obtained by Kaplan-Meier survival analysis and log-rank test was performed to determine statistical significance.

### Bioluminescence imaging

For *in vivo* bioluminescence imaging, mice were anesthetized with isoflurane and injected intraperitoneally with 200 μL of 25 mg/mL D-luciferin sodium salt (Life Technologies). Images were captured with an intensified CCD camera using the Photon Imager (BioSpace Lab). During image acquisition in a light-tight chamber, the animal was maintained on isoflurane via nose cone and body temperature was maintained using a warming pad. Image acquisition times were typically 1 minute. Images were processed by co-registering a reference image with the bioluminescence data image. For *ex vivo* bioluminescence imaging, mice were euthanized and organs were removed immediately and placed into 12well plates. 200-400μL of D-luciferin was added to the organs and the plates were imaged for 1 minute. M3Vision software was used to draw region of interest and to quantify the bioluminescent intensity (count per minute). T-test was performed to determine statistical significance.

### Analysis of *BUB1B* expression data from The Cancer Genome Atlas (TCGA) Research Network

TCGA RNA-seq data were obtained from the Cancer Genomics Hub at UC Santa Cruz and analyzed using HTSeqGenie as previously described [32, 33]. *BUB1B* gene expression level for each sample was quantified in terms of normalized Reads Per Kilobase of exon model per Million mapped reads (nRPKM), defined as number of reads aligning to the *BUB1B* gene / (total number of uniquely mapped reads for each sample normalized by size factor x *BUB1B* gene length). *BUB1B* overexpression (log2 nRPKM) in a tumor sample is defined as an expression level higher than 3 standard deviations above the average expression in 58 solid normal lung tissue samples collected from TCGA patients with lung adenocarcinoma.

Survival analyses were performed with the Kaplan-Meier method and Cox proportional hazards model, using the survival package in the R project for statistical computing. Days to last follow-up or death for lung adenocarcinoma patients were obtained from TCGA's open-access HTTP directory. A multivariate cox proportional hazard analysis was performed to model overall survival in function of *BUB1B* expression status (high nRPKM expression above median, low nRPKM expression below median), smoking history (lifelong non-smoker, current smoker, current reformed smoker), and somatic *KRAS* mutation status (G12 mutation, other protein-altering mutation, wild-type).

To determine *KRAS* and *TP53* mutation status from TCGA, Exome-seq data for lung adenocarcinoma tumor and matched normal (blood or solid tissue) samples were obtained from the Cancer Genomics Hub at UC Santa Cruz and analyzed using HTSeqGenie [32], with Genome Analysis Toolkit (GATK) for variant calling [34]. Tumor-specific somatic variants were obtained by comparing tumor and matched normal samples, excluding common polymorphic variants from the Single Nucleotide Polymorphism database (dbSNP) version 132 [35] that are not reported in the Catalogue of Somatic Mutations in Cancer (COSMIC) database [36].

## SUPPLEMENTARY MATERIAL FIGURES AND TABLES


