# Supplementary material for: Requirement for BUB1B/BUBR1 in tumor progression of lung adenocarcinoma
Source: Genes Cancer. 2015 Mar;6(3-4):106–18. doi: 10.18632/genesandcancer.53 (PMC4426948; doi:10.18632/genesandcancer.53)
Supplement: Supplementary file 1 [file ganc-06-106-s001.pdf]

Requirement for BUB1B/BUBR1 in tumor progression of lung adenocarcinoma

Supplementary Material

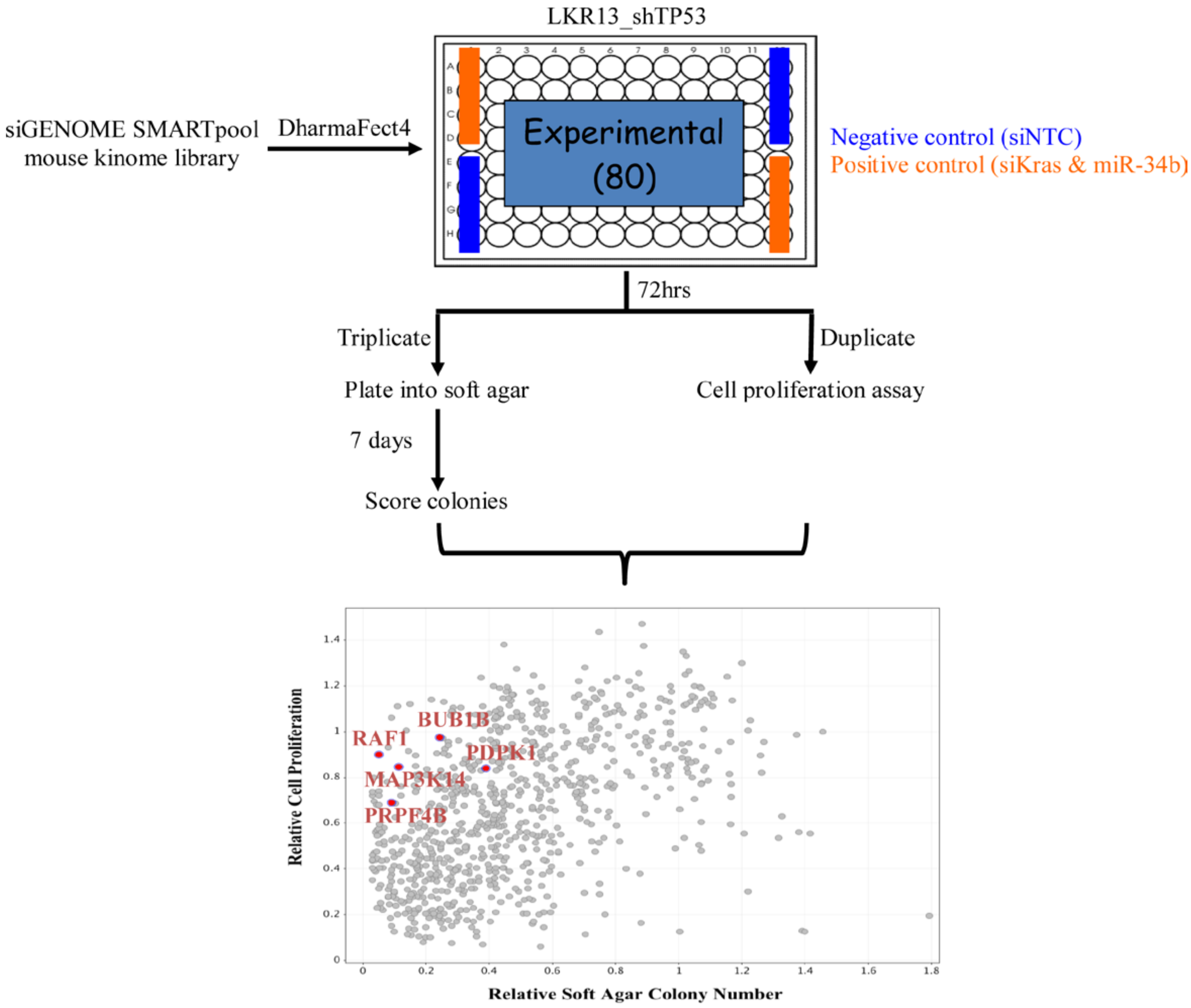

Supplemental Figure S1. Schematic overview of mouse kinome siRNA screen. See Materials and Methods for detailed description. Cell proliferation and soft agar colony numbers were shown as relative values normalized to the siNTC control. The five top hits are highlighted.

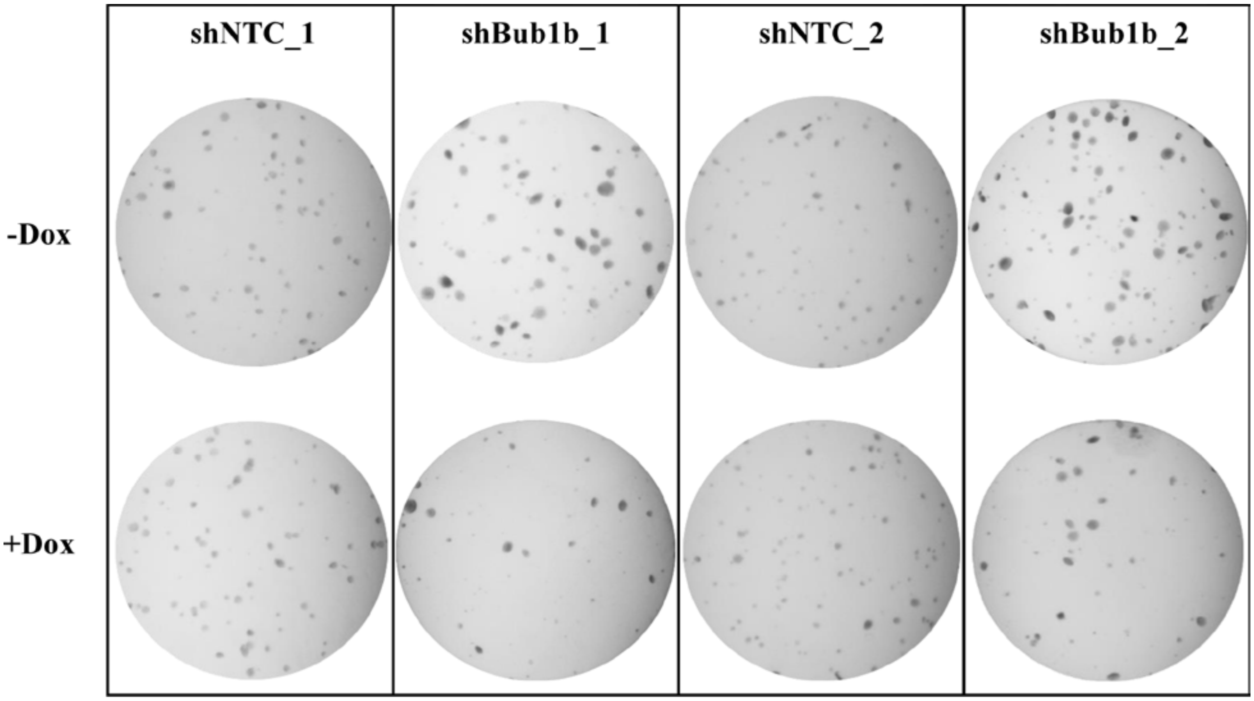

Supplemental Figure S2: Representative images of soft agar colonies formed by LKPH2 stable cell lines expressing the indicated inducible shRNAs.

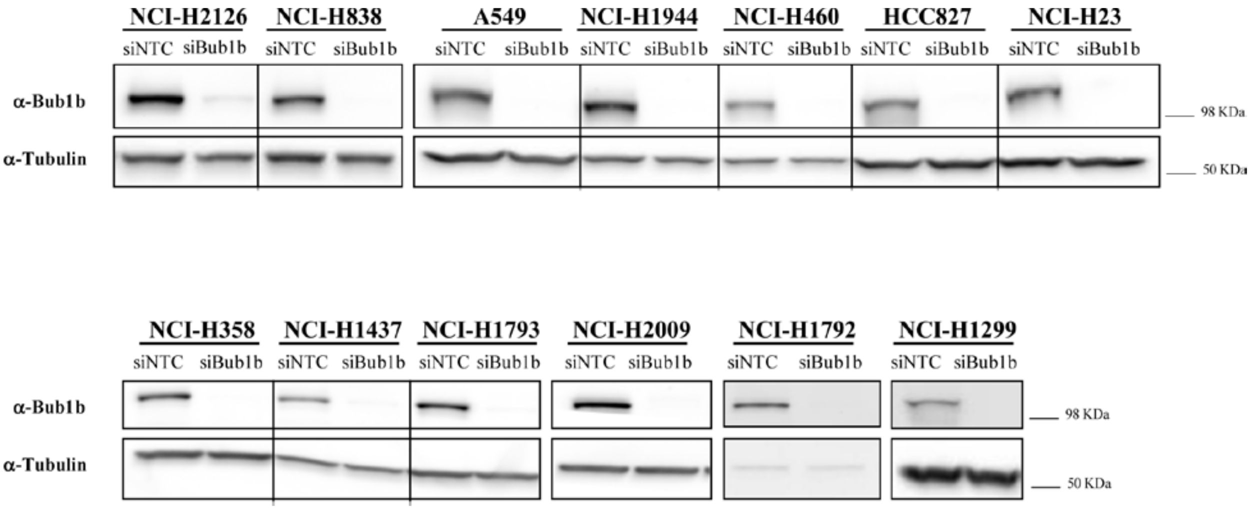

Supplemental Figure S3: BUB1B knockdown in human lung adenocarcinoma cell lines.

Proteins were collected 72 hours after transfection with siNTC or human siBub1b OTP SMARTpool.

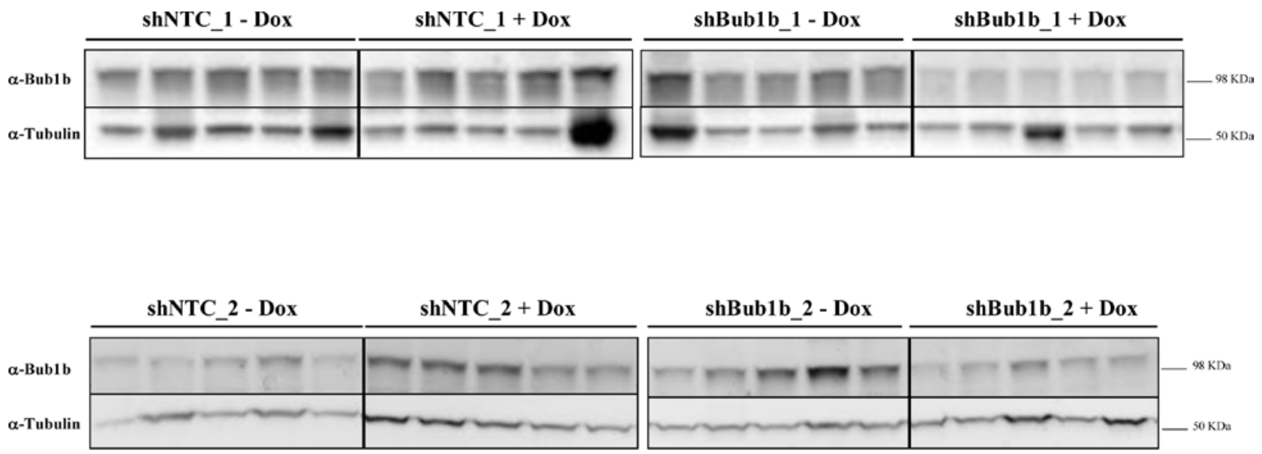

Supplemental Figure S4: BUB1B knockdown in allograft tumors. Tumors were harvested at the end of study. BUB1B protein levels in five tumors from each group are shown.

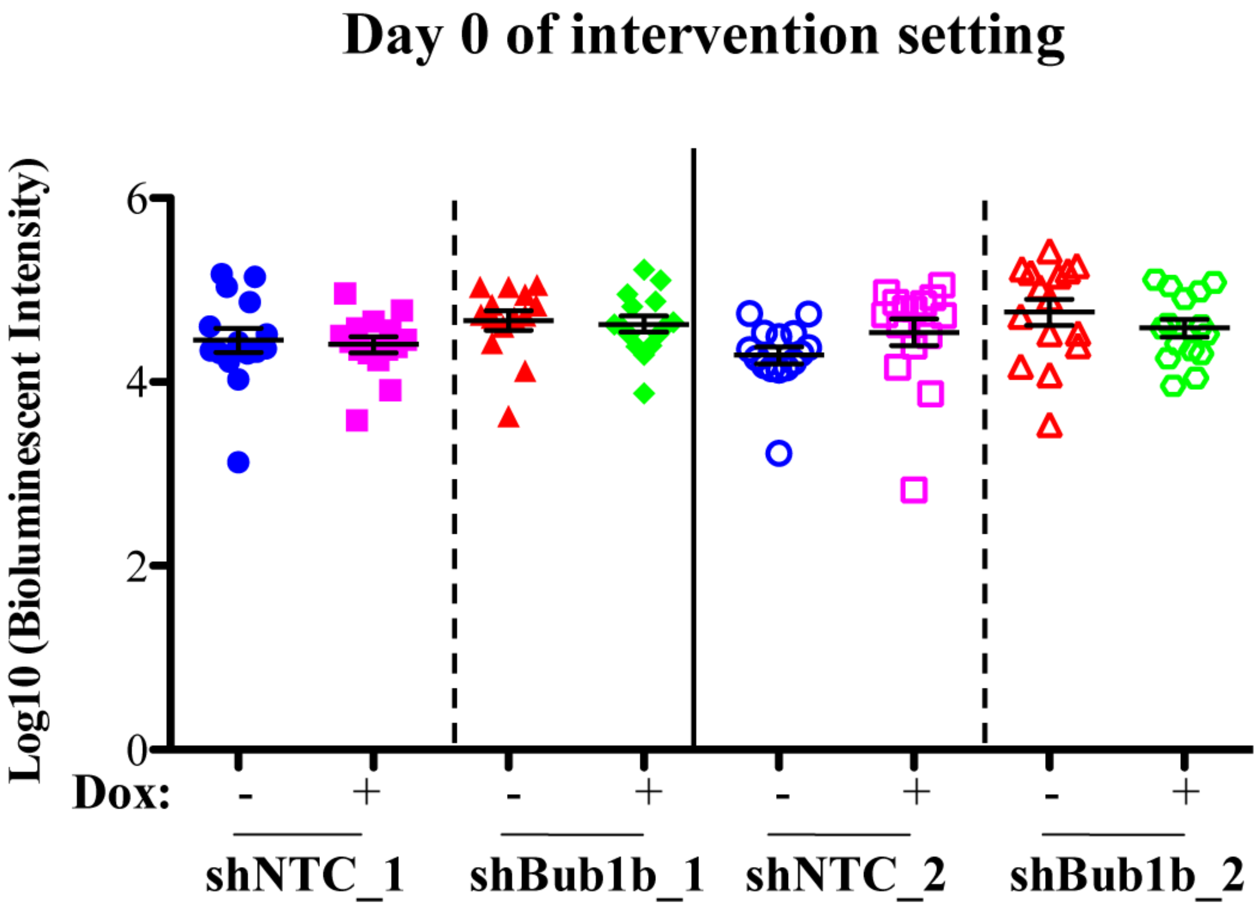

Supplemental Figure S5: Equivalent tumor burden in mice from all experimental groups at the beginning of intervention setting in the tail vein injection mouse model. Quantification of whole body tumor burden by *in vivo* bioluminescence imaging at day 16 post LKPH2 cell injection.

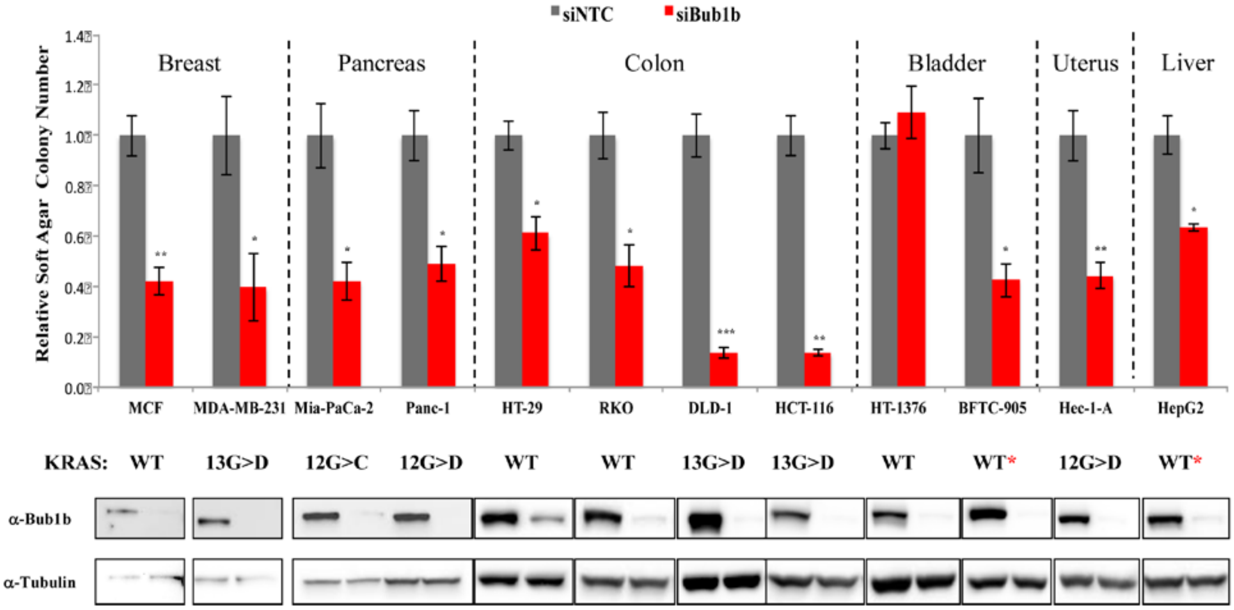

Supplemental Figure S6: Soft agar colony formation and BUB1B knockdown in additional human cancer cell lines transfected with siNTC or human siBub1b OTP SMARTpool. Colony numbers were shown as relative values normalized to siNTC controls. Mean±SEM was calculated from two to three independent experiments. *** indicates p<0.001, ** p<0.01, * p<0.05. Cancer type and *KRAS* status for each cell line are shown above and below each panel, respectively. WT, wild type. A red asterisk indicates the cell line contains *NRAS* 61Q>L mutation. Proteins were collected 72 hours after transfection.

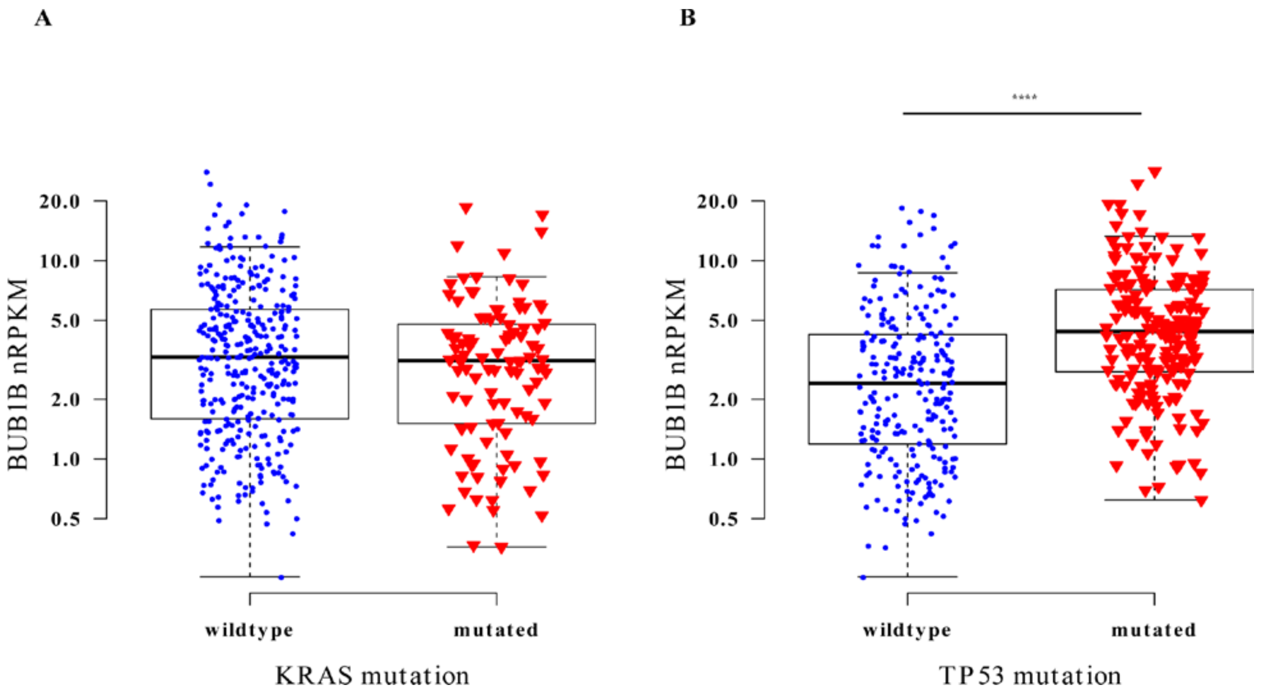

Supplemental Figure S7: Expression of *BUB1B* in TCGA lung adenocarcinoma patients with varied *KRAS* and *TP53* status. Boxplots of *BUB1B* mRNA expression levels in function of *KRAS* (A) and *TP53* (B) mutation status for 448 lung adenocarcinoma samples from TCGA with both RNA-seq and exome-seq data. *KRAS/TP53* mutations only include somatic, protein-altering mutations. **** indicates $p < 0.0001$.

Supplemental Table S1: Lung adenocarcinoma cell lines

Name	Tissue species	KRAS mutation status	TP53 mutation status	Source [reference]
LKR13	Mouse	Mutant 12G>D	Wild type	Lung tumors from *Kras*$^{LSL\text{-}G12D/+}$ mouse [8]
LKPH2	Mouse	Mutant 12G>D	Null[a]	Lung tumors from *Kras*$^{LSL\text{-}G12D/+}$;*Trp53*$^{Flox/+}$ mouse [9]
LKP9	Mouse	Mutant 12G>D	Null	Lung tumors from *Kras*$^{LSL\text{-}G12D/+}$;*Trp53*$^{Frt/Frt+}$ mouse[b]
NCI-H1437	Human	Wild type	Mutant	Genentech Cell Line Repository
NCI-H1793	Human	Wild type	Mutant	Genentech Cell Line Repository
NCI-H2126	Human	Wild type	Mutant	Genentech Cell Line Repository
NCI-H838	Human	Wild type	Mutant	Genentech Cell Line Repository
HCC827	Human	Wild type	Mutant	Genentech Cell Line Repository
NCI-H1299	Human	Wild type	Null	Genentech Cell Line Repository
NCI-H358	Human	Mutant 12G>C	Null	Genentech Cell Line Repository
NCI-H1792	Human	Mutant 12G>C	Mutant	Genentech Cell Line Repository
NCI-H23	Human	Mutant 12G>C	Mutant	Genentech Cell Line Repository
NCI-H2009	Human	Mutant 12G>A	Mutant	Genentech Cell Line Repository
A549	Human	Mutant 12G>S	Wild type	Genentech Cell Line Repository
NCI-H1944	Human	Mutant 13G>D	Wild type	Genentech Cell Line Repository
NCI-H460	Human	Mutant 61Q>H	Wild type	Genentech Cell Line Repository

a. Genomic analysis revealed that the LKPH2 cell line had undergone loss of heterozygosity and become *Trp53* null.
b. Genentech unpublished data

Supplemental Table S2: RNAi target sequences

Name	Supplier	Catalog #	Sequence (sense, 5'→3')
Mouse *Bub1b* siRNA OTP SMARTpool	Dharmacon	L-044095-00-0005	See manufacturer website
Human *BUB1B* siRNA OTP SMARTpool	Dharmacon	L-004101-00-0005	See manufacturer website
OTP non-targeting control (siNTC)	Dharmacon	D-001810-01-50	See manufacturer website
OTP siBub1b_1	Dharmacon	Custom	TAGCAGACCTAGCGCACTT
OTP siBub1b_2	Dharmacon	Custom	AGGATGTGTCTCCCGATATTT
shNTC_1[a]	Genentech	N/A	TTAGTCGACATGTAAACCGCT
shNTC_2	Genentech	N/A	CAACAAGATGAAGAGCACCAA
shBub1b_1[a]	Genentech	N/A	TAGCAGACCTAGCGCACTT
shBub1b_2	Genentech	N/A	AGGATGTGTCTCCCGATATTT
shTP53	Genentech	N/A	CAGTCTACTTCCCGCCATA

a. shNTC_1 and shBub1b_1 share the same hairpin design that is different from the one shared by shNTC_2, shBub1b_2 and shTP53.

Supplemental Table S3: Antibodies

Protein	Supplier	Catalog #
BUB1B	Abcam	ab28193
Flag (clone M2)	Sigma-Aldrich	A8592
α-Tubulin	Sigma-Aldrich	T5168
Mouse IgG HRP conjugated	Amersham	NA931
Rabbit IgG HRP conjugated	Amersham	NA934
Sheep IgG HRP conjugated	Abcam	ab97125
Cleaved caspase-3 (Asp175) (D3E9) Pacific Blue conjugated	Cell Signaling	8788
